# Diversity and Distribution of Terpenoids in Bryophytes and Chemosystematic Uses

**DOI:** 10.3390/plants15132070

**Published:** 2026-07-03

**Authors:** Kakali Sen, Danka Bukvički, Yoshinori Asakawa

**Affiliations:** 1 Department of Botany, University of Kalyani, Nadia, Kalyani 741235, West Bengal, India; 2 Faculty of Biology, University of Belgrade, Studentski trg 16, 11000 Belgrade, Serbia; dankabukvicki@bio.bg.ac.rs; 3 Institute of Pharmacognosy, Tokushima Bunri University, Tokushima 770-8514, Japan; asakawa@ph.bunri-u.ac.jp

**Keywords:** terpenoids, carbon skeleton, diversity, chemotype, biosynthesis

## Abstract

Among the three lineages of bryophytes, liverworts exhibit a wide variety of terpenoid fingerprints. Terpenoids are abundant in the oil bodies of liverworts. Hornworts and mosses are also reported to contain sesqui-, di-, and triterpenoids, although they lack oil bodies. Overall, the abundance of sesquiterpenoids is much greater than that of other types of terpenoid compounds. The occurrence of triterpenoids is very low. Terpenoids found in higher plants are detected in Marchantiophyta in their enantiomeric forms, with a few exceptions. Organic chemists discovered many di- and sesquiterpenoids with interesting carbon skeletons. Bryophytes possess microbial terpene synthase-like enzymes that are different from typical plant terpene synthases. Original research articles and high-quality reviews were extracted from Google Scholar, PubMed, ScienceDirect, and Scopus using the keywords “terpenoid diversity”, “terpenoid and chemosystematics”, “terpenoids of bryophytes”, “oil bodies”, “terpene synthase”, “microbial terpene synthase-like enzymes”, and “genomics and terpenoid research progress” to prepare this review. Only the literature published in the English language was considered. This review focused on the terpenoid diversity of bryophytes, including chemosystematics uses, as well as the varying carbon skeletons of terpenoids. Oil body biogenesis and evolution, along with the terpene biosynthesis pathway and related enzymes, are briefly covered. The emergent area of multi-omics approaches may bridge the gap that exists in this field and will also open up future avenues for the use of *Marchantia polymorpha* or *Physcomitrella patens* as an efficient tool for specific and valuable terpenoid production.

## 1. Introduction

Spore-producing bryophytes first colonized land 450 MYA and radiated into three major lineages with diverse body plans, specific morphological organizations, and biochemical signatures [1,2]. Liverwort diversity is represented by ~9000 species, mosses by ~12,700 species, and hornworts by ~250 species [3]. Of the three lineages, Marchantiophyta (liverworts) evolved from marine algae, as proven by their common chemical signature [4]. Greek *Fossombronia angulosa* (Dicks.) Raddi and Tahitian *Chandonanthus hirtellus* (Web.) Mitt. produce similar types of acetogenins, such as brown algae *Dictyopteris* sp. This chemical signature supports the position of liverworts as the basal-most group of extant land plants, and molecular analyses also corroborate this [5].

The selective forces influencing bryophyte evolution following land colonization have been investigated through critical analyses of major morphological, anatomical, and physiological innovations. Furthermore, plastid DNA markers (*rps4*, *trnL–F*, and *rpl16*) [6,7] and plastome sequences have contributed to the reclassification of bryophytes [8]. In addition, secondary metabolites in these organisms provide protection and have become an invaluable chemosystematic attribute for species assignment. The presence of oil bodies and the accumulation of specific types of secondary metabolites within have interesting eco-evolutionary significance. Oil bodies in liverworts (except the genus *Metzgeria*) separate them from their sister lineage/mosses and possess interesting biological activities [9,10,11,12]. One of the predominant secondary metabolites of oil body cells is terpenoids. Approximately 1600 types of terpenoid compounds are reported from liverworts [13]. Terpenoids play an important role in plant photosynthesis by regulating plant growth and development, pollination, and resistance to environmental stress. Terpenoids are even key players in herbivore and pathogen defense, as they are secreted for natural defense when the plant is eaten by pests. Volatiles released by plants attract natural enemies of the pest, achieving pest control. On the other hand, protecting the plants themselves can also have an impact on the environment, and these compounds influence the evolution of plant communities and ecosystems. Terpenoids are the most important cue in regulating this tritrophic relationship of the plant with pests and their natural enemies [14]. Terpenoids are built from isoprene (C_5_H_8_) units. Two units form monoterpenoids, three form sesquiterpenoids, four form diterpenoids, and additional units are joined to form higher terpenes. Norterpenes are formed by one or more carbon losses from the parent skeleton in the respective group. Steroids are reported and found to be formed from triterpenoids. The chemical constituents help to delineate taxa and establish evolutionary relationships at different taxonomic hierarchies to distinguish intergeneric and interfamilial distinctions. Furthermore, secondary metabolite biosynthesis in bryophytes is influenced by several factors, such as the developmental stage, season, altitude, and sexuality of the plant, thereby contributing to assessments of reproductive fitness and population differentiation [15,16]. The diversity of secondary metabolites was explored from different countries and continents; chemotypes were reported; and characterization, synthesis, and bioactivity were also explored [17,18,19]. Essential oils and volatiles in bryophytes were investigated and reviewed earlier [13,20,21,22].

**Figure 1:**
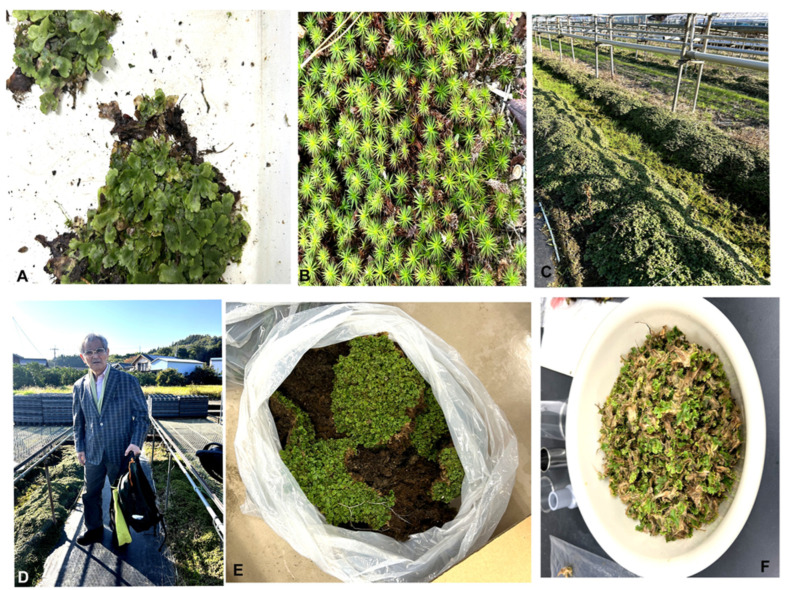
(**A**) *Conocephalum conicum* (from India), (**B**) *Polytrichum commune* (Ritsurin Garden, Japan), and (**C**–**F**) *Marchantia polymorpha* collection during field surveys led by Prof. Asakawa (Tokushima, Japan).

To prepare this review, original research articles and comprehensive review papers were systematically collected from Google Scholar, PubMed, ScienceDirect, and Scopus. The literature search was conducted using the keywords “terpenoid diversity”, “terpenoids and chemosystematics”, “terpenoids of bryophytes”, “oil bodies”, “terpene synthase”, “microbial terpene synthase-like enzymes”, and “genomics and terpenoid research progress”. Only studies published in the English language were included. This review summarizes the recent updates on the terpenoid diversity of bryophytes, the presence of enantiomeric components, with a special emphasis on terpenoids with unusual carbon skeletons, and their role in the study of chemosystematics. Terpene biosynthesis and recently discovered related genes are covered briefly. In addition, a critical analysis has been provided to analyze the existing knowledge gap, which can lay the foundation for future research questions. Figure 1 shows liverwort collections in India and Japan by the authors of this article for terpenoid characterization.

## 2. Oil Bodies—Biogenesis and Evolution

Oil bodies are single-unit membrane-bound intracellular organelles containing lipophilic compounds suspended in a proteinaceous matrix [23]. Terpenoids (e.g., sesquiterpenes) and aromatic compounds (bibenzyl acids) are the main components of oil bodies, with direct evidence for sesquiterpene-thujopsene, *β*-chamigrene, and bisbibenzyl compound marchantin A [24,25,26]. The accumulation of such chemicals can be highly toxic to the plant itself, so these organelles (oil bodies) are required to avoid self-toxicity [26].

The presence of oil bodies in liverworts was reported much earlier, in 1834 by Huebner, from leafy liverwort *Jungermannia taylorii* (now *Mylia taylorii*), and confirmed later on through the aid of light and electron microscopy by Pihasaki. Oil bodies are active cell compartments and are the site of isoprenoid synthesis in liverworts [9]. Significant variation in the size, number, and chemical composition of oil body cells is recorded in different taxa of liverwort. The oil bodies of *Radula constricta* are large in size and present in all cells, while *Trocholejeunea sandvicensis* has dozens of small oil bodies. Numerous small and homogeneous oil bodies in *Austrofossombronia*, large oil bodies in *Calypogeia*, large solitary papillose oil bodies in *Jungermannia,* and large, solitary oil bodies are found in specialized idioblast cells in *Marchantia polymorpha* [27,28]. However, some liverworts, such as *Riccia crystallina* and *Anthelia julacea,* do not possess any oil bodies. A fluorescent dye, Bodipy, is used to stain oil body cells [28]. Nile red staining of the oil body suggests that an energy reserve could be accumulated within them in the form of lipids [29]. The biogenesis of oil bodies is subject to several hypotheses. They either form from vacuole-like structures, into which the ER or Golgi apparatus secretes substances, such as small droplets, or they are developed from the fusion of cytoplasmic lipid droplets or from dilated cisternae [9]. One of the key enzymes of sesquiterpenoid biosynthesis is isoprene synthase, localized in the oil body membrane [30]. MpSYNTAXIN OF PLANTS 12B (*SYP12B*) is detected in *Marchantia polymorpha* in the membrane of the oil body, and it was suggested that the formation of the oil body is analogous to cell plate formation [31]. Transcriptional regulation of oil body formation has been studied, and Kanazawa et al. demonstrated that ERF/AP2-type transcription factors act as master regulators of oil body formation. In *M. polymorpha,* the MpERF13 knock-out mutant shows a complete lack of oil bodies, while the gain-of-function mutant overexpresses the MpERF13 gene, resulting in the increased production of oil bodies [11]. Similarly, MpC1HDZ, a class I homeodomain leucine-zipper transcription factor, acts as another key regulator in oil body formation, as proven through a mutant study [12]. A transcription factor, i.e., MpTGA from the basic leucine-zipper (bZIP) family, functions as a negative regulator of oil body formation, as the knock-out mutant shows upregulation of key oil-body-associated genes. Vesicle-mediated trafficking plays a key role in oil body biogenesis. An active vesicle-mediated assembly mechanism that mediates oil body formation is evidenced and supported by several studies [30]. The exclusive exocytic or endocytic origin of the oil body was not confirmed. Involvement of the secretory pathway in oil body formation was supported by a transgenic line, and one secreted marker, sec-mRFP, was targeted to the extracellular space in non-oil-body cells. However, sec-mRFP was found to accumulate inside oil body cells. Vesicles typically destined for secretion are redirected to participate in oil body formation, indicating a rerouting of the secretory pathway for oil body formation [11]. Involvement of transcription regulation in the secretory pathway was found. *MpSYP12 B*, *MpSYP13A*, and *MpSYP13B* play critical roles, as their localization is regulated at the transcriptional level and would determine intracellular trafficking dynamics within the oil body cells [11].

Liverworts possess unique oil bodies in their cells as a synapomorphy during land plant evolution, which were previously supposed to provide protection against desiccation and increased UV radiation, although experiments with transgenic lines of *Marchantia* lacking oil bodies have shown that these plants are able to grow under abiotic stress conditions, such as high salinity, osmotic stress, and desiccation. Instead, the plants become more susceptible to herbivorous insects, supporting an ecological defense role [12]. Using oil body characteristics, a synthetic phylogeny of liverwort was reconstructed, depicting the relationship of liverwort lineages, i.e., Haplomitriopsida, Jungermanniopsida, and Marchantiopsida. Reconstruction reveals that oil bodies are present in the most common recent ancestors of liverworts, and a major macroevolutionary pattern was predicted, although the ancestral condition needs to be supported by sampling more taxa [29]. The chemical diversity of the oil body cells was explored, and 3000 specialized metabolites have been described from the three major lineages, of which 1600 terpenoids with bibenzyls and bisbibenzyls represented the most prominent aromatic compounds [13].

## 3. Chemosystematics of Bryophytes Using Terpenoids

Three lineages of bryophytes, i.e., liverworts, mosses, and hornworts, were used, which differ markedly in morphological traits, the presence or absence of water-conducting strands, spore germination patterns, and chemical composition. Chromatographic fingerprinting may be used as an additive tool to morphological and genetic data for resolving taxonomic issues at the species, genus, and family levels [32]. Chemosystematic studies have significantly contributed to resolving taxonomic relationships among bryophytes. For instance, members of the order Jungermanniales are characterized by the presence of pinguisane-type sesquiterpenoids, which also occur in the genus *Aneura* of the order Metzgeriales. This shared chemical signature strongly suggests that Jungermanniales and Metzgeriales diverged from a common ancestor [4]. Geographical variation in secondary metabolite composition often results in the formation of distinct chemotypes. Multiple chemical races have been reported across liverwort families. Volatile profiling of *Radula* species from Portugal revealed two distinct chemotypic clusters based on sesquiterpene composition, highlighting geographical variation within the genus [33].

Species such as *Makinoa crispata*, *Metzgeria furcata var. furcata*, and *Jungermannia infusca* each exhibit three chemotypes, whereas *Scapania undulata*, *Lepidozia vitrea*, *Reboulia hemisphaerica*, and *Conocephalum conicum* have been characterized by four, two, three, and three chemical races, respectively [4,34,35,36]. Chemotypes of *Frullania* sp. are characterized by the presence of terpenoids and aromatics [37]. Extensive chemical surveys of *Frullania* species from New Zealand, Australia, and South America led to their classification into five major chemotypes: sesquiterpene lactone, sesquiterpene lactone–bibenzyl, bibenzyl, 2-alkanone, and triterpene types. The closely related *Schusterella chevalierii* exhibits chemical similarity to the sesquiterpene lactone type by producing eudesmanolides, β-cyclocostunolide, and dihydro-β-cyclocostunolide [38]. Chemical investigations of *Riccardia* species from Europe, Japan, Taiwan, and South America revealed sesquiterpenoids and aromatic compounds as the main metabolites. Ether extracts of Malaysian liverworts revealed herbertane-, gymnomitrane-, chiloscyphane-, eudesmane-, germacrane-, and guaiane-type sesquiterpenoids as chemotaxonomic markers. The characteristic fragrance of *Wiesnerella denudata* and *Dumortiera hirsuta,* belonging to the order Marchantiales, is due to the presence of monoterpenoids [5]. Different chemotypes of *Conocephalum conicum* possess at least 12 monoterpenoids, including unique bornyl cinnamate esters such as (+) bornyl p-coumarate and (+) bornyl ferulate [4,39,40,41].

The primarane diterpenoids momilactone A and B, known phytoalexins from rice, were reported later from *Hypnum plumaeforme* (a moss), where they function as allelochemicals [42]. While these compounds are synthesized from geranylgeranyl diphosphate in rice, their biosynthetic pathway in mosses remains unresolved. The occurrence of drimanes in *Porella vernicosa*, *Makinoa crispata*, and the New Zealand fern *Blechnum fluviatile*, as well as phenylbutenones in *Hymenophyton flabellatum* and the fern *Arachinoides standishii*, provides chemical evidence supporting evolutionary links between hepatics and pteridophytes [43,44]. Some more taxa with distinct terpenoid profiles used for chemosystematics are presented in Table 1. Given the unique phytochemistry of liverworts, chromatographic fingerprinting offers valuable insights into phylogenetic relationships at the species level and higher taxonomic levels [32]. While molecular approaches based on gene sequencing are now routinely used to resolve phylogenetic relationships due to limitations in traditional morpho-taxonomy [45], reliance on molecular tools alone risks diminishing expertise in classical taxonomy. A holistic approach integrating morphology, anatomy, and chemosystematics is therefore essential for robust bryophyte classification. Overall, surveys of chemical races indicate that the occurrence of multiple chemotypes within a single species may arise from geographical variation or population-level differences. Consequently, expanded chemical investigations across diverse geographical regions are imperative to fully understanding intraspecific variation, evolutionary relationships, and the chemical diversity of bryophytes.

## 4. A Comparative Account of Terpenoid Diversity in Bryophytes: Evolutionary Significance and Varying Carbon Skeletons

**Figure 2:**
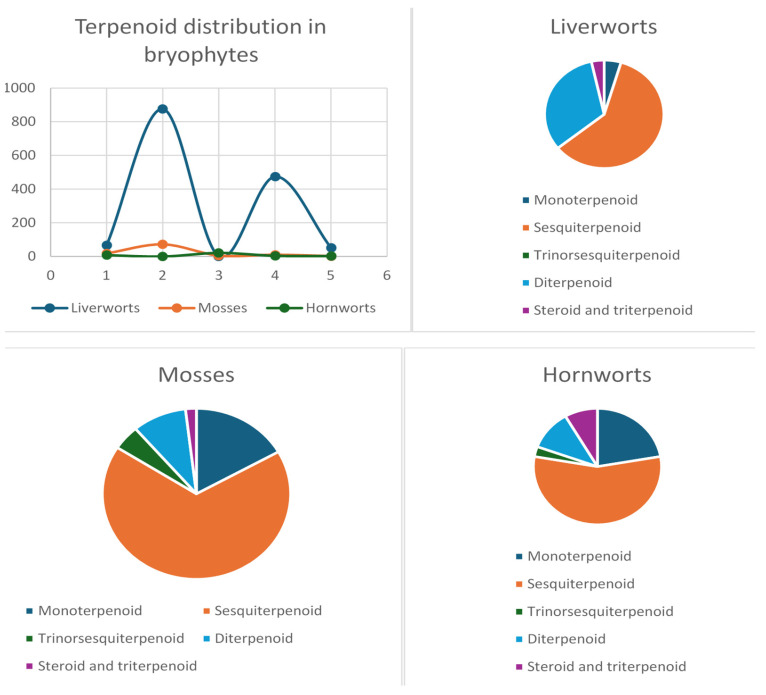
The line diagram (**upper left**) shows the distribution of terpenoids in three major lineages of bryophytes. The rest of the pie chart shows different classes of terpenoid compounds in the respective classes, such as liverworts (**upper right**), mosses (**lower left**), and hornworts (**lower right**). Trinorsesquiterpenoids have not yet been reported from liverworts.

Bryophytes are a treasure house of terpenoid compounds. About 1600 terpenoid molecules have been discovered. Chirality and unusual asymmetric carbon centers contribute to the rich profile of terpenoid compounds. Asakawa and his co-workers studied the terpenoid chemistry of bryophytes in a very extensive way using a worldwide, diverse collection pool. For a detailed distribution of terpenoid compounds across taxa, readers are referred to reviews [13,50]. The following account describes a brief comparison of the terpenoid diversity of liverworts, hornworts, and mosses, and the major trend of evolution in three lineages of bryophytes, emphasizing unusual and novel carbon skeletons. Different terpenoid classes, such as mono-, norsesqui-, sesqui-, di-, and triterpenoids, as well as steroids, are reported from Marchantiophyta, Bryophyta, and Anthocerotophyta [4]. The frequency of distribution of terpenoids and terpenoid subclasses is shown in Figure 2.

### 4.1. Monoterpenoid Distribution and Diversity

**Figure 3:**
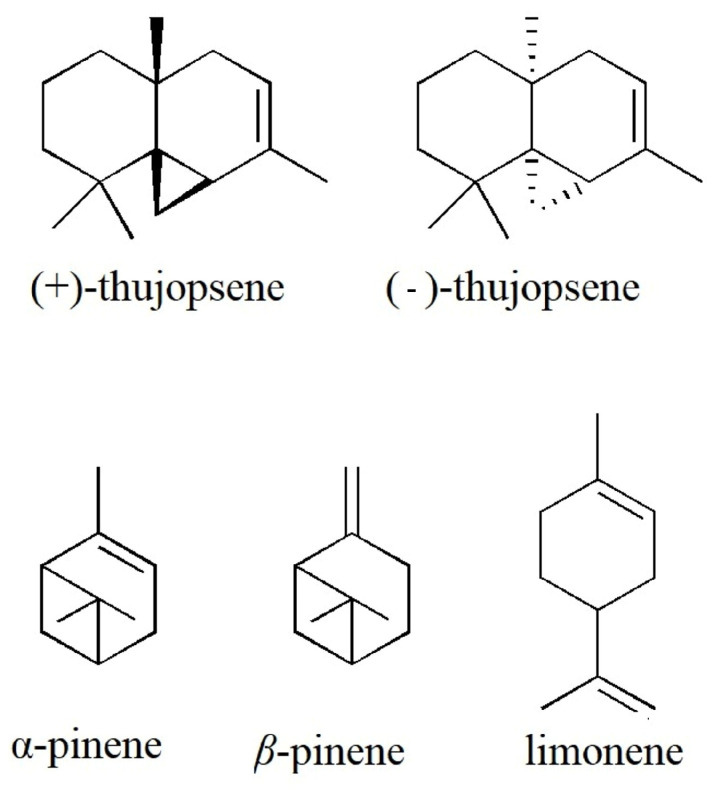
Some of the volatile terpenoids present in liverworts.

Acyclic to mono-, bi-, and tricyclic monoterpenoid compounds are detected in Marchantiophyta. In most cases, hydridistillation techniques were used, followed by characterization via GC/MS. A total of 67 monoterpenoids have been discovered from liverworts, of which the first isolated was β-cyclocitrol from *Jungermannia hattoriana*. Monoterpenoids are responsible for pleasant or unpleasant odors (see Table 2). Some liverwort species release a strong aromatic odor when their tissues are crushed. This odor is usually associated with the presence of volatile monoterpenoids. Previous investigations have demonstrated that such compounds are particularly common in liverworts belonging to the order Marchantiales, which comprises complex thalloid species. Typical fragrant constituents include α-pinene, *β*-pinene, and limonene (Figure 3) [51,52,53,54,55].

Enantiomeric counterparts of liverwort monoterpenoids are reported from higher plants, with some exceptions [46]. The monoterpenoid diversity of mosses is much less than that of liverworts. Monoterpenoids are found to occur as volatile constituents of essential oils in mosses. Common constituents include myrcene, limonene, α- and β-pinene, camphene, terpinene-4-ol, α-terpineol, borneol, bornyl acetate, and camphor. Cyclocitral was detected frequently across several species (Figure 3). Apart from camphor, pinocarvone, and carvone, most monoterpenoids identified in these mosses are also known to be present in liverworts, indicating a shared monoterpenoid diversity among bryophytes [50].

*Anthoceros caucasicus* from Maderia was investigated using GC-MS fingerprinting, and the presence of *β*-myrcene, *γ*-terpinene, terpinolene, limonene, p-cymene, α-pinene, β-pinene, and camphene was reported. Limonene was the major component of the reported compounds [56]. Furthermore, the presence of mono- and sesquiterpenoids in two additional hornwort species, *Anthoceros punctatus* and *A. agrestis*, was confirmed [57].

### 4.2. Norsesquiterpenoid and Sesquiterpenoid Diversity

**Figure 4:**
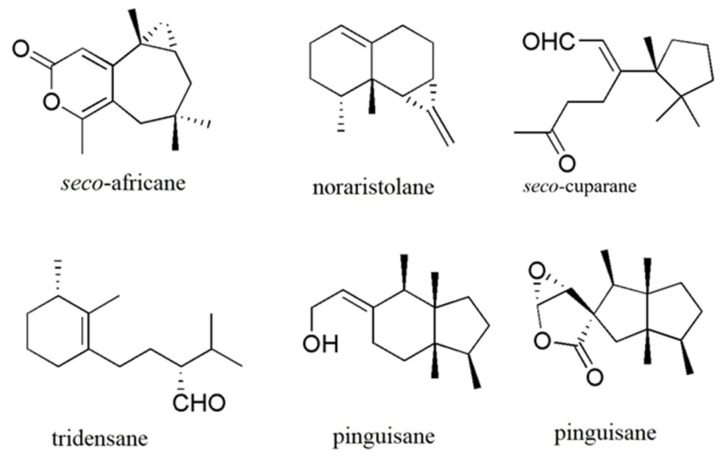
Some characteristic sesquiterpenoids present in liverworts.

**Figure 5:**
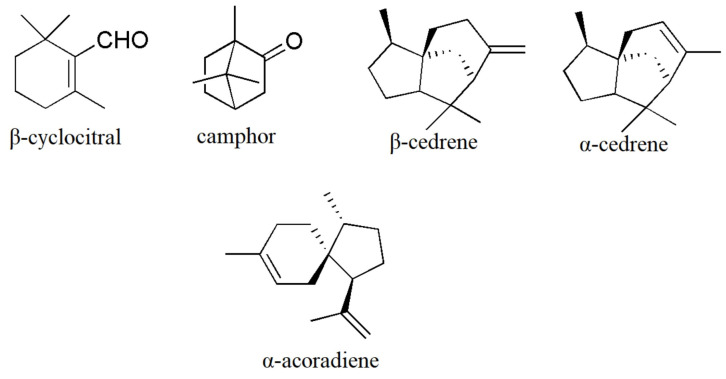
Some characteristic sesquiterpenoids present in liverworts and mosses.

Sesquiterpenoids represent the most diverse subclass of terpenoids in Marchantiophyta (liverworts) and Bryophyta (mosses) (Figure 4 and Figure 5). To date, approximately 45 different sesquiterpenoid carbon skeletons have been identified, many of which are rare in nature, while others occur as enantiomers of compounds found in higher plants. However, trinorsesquiterpenoids have not yet been reported from liverworts. Several sesquiterpenoid types are widely distributed and abundant among different liverwort taxa [50].

Sesquiterpenoids are less volatile than monoterpenoids, with acorane-type sesquiterpenoids being characteristic types. The genus *Radula* is a major source of acorane sesquiterpenoids. *Radula aquilegia*, *R. complanata*, *R. perrottetii,* and *R. wichurae*, collected mainly from Japan and East Asian regions, contain 2–5 sesquiterpenoids per species, including α- and β- acoradiene and related derivatives (Figure 5) [33,58,59]. Acorane and africane skeletons are relatively rare in liverworts. Other than *Radula* spp., acoranes are reported from *Jungermannia hattoriana* and *Barbilophozia hatcheri*. Africane-type sesquiterpenoids have been detected in Canadian and Japanese populations of *Herbertus aduncus*, as well as in *Pellia epiphylla* and *Porella subobtusa* [60]. Trinoraromadendrane derivatives are also extremely uncommon in nature; *Bazzania praerupta*, *Barbilophozia floerkei*, and *Lophozia ventricosa* are known to produce trinoranastreptene [61].

The occurrence of azulenoids in liverworts is restricted to a few species of *Calypogeia* and *Plagiochila*. Barbatane (gymnomitrane) sesquiterpenoids were first discovered in liverworts, and their enantiomers were subsequently identified in the higher plant *Meum athamanticum* by König and co-workers [62]. Bazzanane-type and bergamotane sesquiterpenoids are also rarely encountered in nature. Among sesquiterpene hydrocarbons, bicyclogermacrene is one of the most abundant compounds and serves as an important biosynthetic precursor for numerous cyclic sesquiterpenoids. Cadinanes and their stereoisomeric counterparts, the amorphanes, are widely distributed in liverworts. Farnesanes and pinguisanes constitute common sesquiterpenoid types in these plants, whereas thujopsanes are only rarely reported.

Sesquiterpenoid diversity in moss is lower than in liverworts. Some early reports include the isolation of a sesquiterpenic alcohol, myliol, from *Mylia taylorii* [63]. Subsequently, α- and β-barbatane, representing a novel tricyclic sesquiterpene skeleton (gymnomitrane type), were identified from *Barbilophozia* species, along with calamenene and α-alaskene [64]. Studies on *Conocephalum conicum* revealed cadinene-type sesquiterpenes, with *δ*-cadinene occurring as the enantiomeric form found in vascular plants [65]. During the same period, European *Scapania* species were reported to contain enantiomeric humulene–longifolene and germacrene-derived sesquiterpenes, including (+)-*α*- and *β*-chamigrene in *S. undulata* [66]. Japanese *Plagiochila* species were found to contain plagiochiline A, a pungent ent-secoaromadendrane-type sesquiterpene hemiacetal, along with related compounds such as plagiochilide and furanoplagiochilide A [67].

Sesquiterpenoids show remarkable structural diversity in bryophytes. In addition to regular sequiterpenes, trinorsesquiterpenoids, such as ionones and geosmin, were identified from *Plagiothecium undulatum*, *Mnium hornum,* and *Homalia trichomanoides*. Ent-*β*-cedrene, the first sesquiterpenoid reported from mosses, was isolated from *Plagiomnium acutum* [68]. The Japanese moss *Plagiomnium acutum* produces several ent-sesquiterpene hydrocarbons, including *β*-cedrene, α-cedrene, and α-acoradiene (Figure 5) [68]. Rare compounds, such as peculiaroxide, previously known only from liverworts, were also detected, emphasizing shared types of terpenoids between bryophyte lineages. Aromadendrene and a-Barbatene are found in mosses.

**Figure 6:**
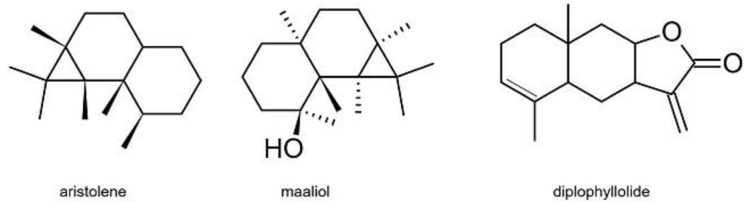
Terpenoids in hornworts.

The trinorsesquiterpene 4,8a-dimethyl-4a,5-epoxydecalin; the sesquiterpene hydrocarbons aristolene, calarene, anastreptene, viridiflorol, palustrol, spathulenol, allo-aromadendrene, α-barbatene, β-barbatene, *β*-bazzanene, *β*-elemene, *β*-bisabolene, *β*-sesquiphellandrene, and *δ*-cuprenene; the sesquiterpene alcohols rosifoliol, (E)-nerolidol, maaliol, and 5-guaiene-11-ol; the sesquiterpene lactone diplophyllolide; and a sesquiterpene ether, veticadinoxide, were reported from *Anthoceros caucasus*. Although the absence of oil bodies suggests the limited presence of essential oils, *A. caucasus* possesses a large amount of cadinene-type sesquiterpene ethers [56]. The essential oil also contained several sesquiterpenoids, including aristolene, maaliol, and diplophyllolide (Figure 6) [56].

### 4.3. Diterpenoids

Diterpenoids are widely distributed in liverworts, with approximately eighteen distinct carbon skeleton types identified to date and 474 diterpenoid compounds. Among these, clerodane diterpenoids are particularly abundant, making liverworts an important natural source of this class of compounds. In contrast, the occurrence of halimane diterpenoids is restricted to a limited number of species within the order Jungermanniales [50]. Species of *Jungermannia* are especially rich in ent-kaurane diterpenoids, which are considered characteristic chemotaxonomic markers of the genus. Members of this genus are noteworthy from both morphological and chemical perspectives because they exhibit considerable polymorphism, and their chemical profiles often vary according to the geographical location of the collection site. Pimarane diterpenoids are rarely encountered in liverworts. Notable examples include ent-pimara-8(14),15-dien-19-ol (1325a) and ent-8-hydroxypimar-15-ene (1325b), both of which have been isolated from *Jungermannia thermarum* [69]. *Marchantia* and *Jungermannia* are reported for diterpenoid diversity, though it is less diversified than monoterpenoids. Diterpenoids are less widespread but chemically significant in bryophytes. Fractionation of *Plagiomnium acutum* (Japan) led to the isolation of (+) dolabella-3,7-diene-18-ol, representing the first dolabellane diterpenoid reported from mosses. Other diterpenoids detected include kaurane, pimarane, abietane, and labdane-related structures, such as 16-kaurane, sandaracopimaradiene, abietatriene, and manool. The occurrence of ent-diterpenoids in mosses parallels liverwort chemistry, suggesting convergent or conserved biosynthetic pathways despite major morphological differences [68]. The diterpenoids ent-16-kaurene and isoabienol have also been detected in the essential oil of *A. caucasicus* [56].

### 4.4. Triterpenoids and Sterols

Triterpenoids are very limited in their presence. They occur in liverworts in trace amounts or as single compounds and are only reported from European and Asian samples. No liverwort species is characterized by a rich triterpenoid profile comparable to that of higher plants. Liverwort terpenoids show chirality and frequent enantiomeric specificity. The triterpene alcohol zeorin is one of the chemical markers of the genus *Plagiochasma* [46]. Triterpenoids and steroids are relatively rare but structurally diverse in bryophytes. Studies revealed dammarane, hopane, serratane, and sterol skeletons in selected moss species. Examples include dammara-(17Z), 21-diene from *Floribundaria aurea* subsp. *nipponica* (Japan/China), hopane derivatives from *Weymouthia mollis*, and serratane triterpenoids from the Chinese moss *Homalia trichomanoides*. Several of these triterpenoids were previously known from liverworts or higher plants, again highlighting chemical continuity across plant lineages.

Triterpenoids are absent in hornworts, although *Megaceros flagellaris* possesses sitosterol, stigmasteryl palmitate, and sitosteryl palmitate, of which sitosterol is the major one [70].

### 4.5. Significance of Chirality and Enantiomers

Liverwort terpenoids exhibit an exceptionally broad diversity of carbon skeletons, despite the relatively simple morphology of these plants. Monoterpenoids encompass acyclic to tricyclic frameworks, including ocimene, *p*-menthane, pinane, camphane, bornane, and thujane skeletons. Sesquiterpenoids further expand this structural diversity, with acorane, alskane, cuparane, and rearranged carbon skeletons being particularly characteristic of liverworts. Acorane-type sesquiterpenoids are especially significant as they are rare in higher plants but widely found in liverworts, pointing to the unique biosynthetic capacity of these groups. In contrast, other types of terpenoids, i.e., di- and triterpenoids, are represented with a limited number of carbon skeletons. The wide range of carbon skeletons is formed due to oxygenation and rearrangements, and produces structurally novel terpenoids within the plant kingdom [4,33,59,71]. The consistent presence of highly enriched enantiomers or optically pure compounds indicates a stereochemically strict terpene synthase, reflecting a high degree of enzymatic control in liverwort metabolism [20]. Chirality is closely linked to chemotypic variation, reflecting differences in the underlying terpene biosynthetic machinery. Distinct chemotypes within a single species often differ not only in their dominant terpenoids but also in the absolute configurations of these compounds. Chemotype-dependent enantiomeric patterns have been observed in liverworts producing borneol, bornyl esters, and acorane-type sesquiterpenoids, where the absolute configuration was shown to be consistent within a chemotype but variable between populations or geographic origins [41,72]. This tight association between chirality and chemotype highlights the importance of stereochemistry as a chemotaxonomic marker in liverworts, emphasizing that chemotypic differentiation not only changes the compound profiles but may also involve fundamental shifts in stereochemical biosynthesis.

An interesting structural feature of many liverwort terpenoids is their stereochemistry. In numerous cases, sesquiterpenoids and diterpenoids isolated from liverworts are enantiomers of compounds known from higher plants, although exceptions occur within certain structural classes such as germacranes and guaianes. For example, the liverwort *Reboulia hemisphaerica* produces (+)-thujopsene, whereas the (−)-enantiomer is a major constituent of cedarwood essential oil (Figure 4).

Chirality is a defining characteristic of bryophyte terpenoids. Several sesquiterpenoids occur as enantiomers distinct from those found in higher plants. A striking example is ent-β-cedrene, isolated from *Plagiomnium acutum*, which was shown by enantioselective GC and optical rotation measurements to be the mirror image of commercially available β-cedrene derived from higher plants. This represented the first report of an ent-sesquiterpenoid in bryophytes and the first record of ent-cedrene in the plant kingdom. Further examples include optically active murolene, cuparenone, and daucane derivatives whose absolute configurations were rigorously established using NMR spectroscopy, NOESY experiments, chemical correlation, and enantioselective GC [68].

## 5. Terpenoid Biosynthesis

Experimental investigation shows that the mevalonate (MVA) and methylerythritol phosphate (MEP) pathways are operative in liverworts for terpenoid production [73]. MVA and MEP pathways are the main sources of the C_5_ precursor unit, consisting of two isomers, i.e., IPP and DMAPP. The MVA pathway includes six enzymatic reaction steps, providing precursors for sesquiterpenes and triterpenoids. The seven enzymatic steps in the MEP pathway mainly act as substrate sources for monoterpenes and diterpenes [14]. After the formation of precursors, the downstream steps of terpene formation required process modification, which regulates rich arsenals of terpenoid compounds. Following the synthesis of the terpenoid carbon skeleton, terpenoids can be further modified by redox reactions, methylation, acetylation, and glycosylation by cytochrome P450(CYP) and other modifying enzymes, giving complex and diverse chemical structures to terpenes [14]. Sabinene synthase and bornyl diphosphate synthase were purified and characterized from *Conocephalum conicum*. The cyclization of geranyl diphosphate to sabinene required one monoterpene cyclase, which was purified and characterized as a soluble enzyme of Mr 65,000, with an optimum pH of 7.5 and Mg^2+^ as a cofactor. It resembles other monoterpene cyclases from higher plants. The bornane-type monoterpene is derived from geranyl diphosphate as in higher plants, through the action of bornyl diphosphate synthase [74].

The mevalonate pathway (MVA) is found in eukaryotes, archaea, and some bacteria. In plants, the first steps occur in the cytosol, although the final steps occur in the peroxisome. Immunolabeling experiments in *Marchantia* cells show that key enzymes are compartmentalized in the oil bodies, chloroplasts, and cytosol, or in several compartments simultaneously. 1-deoxy-D-xylulose-5-phospahate synthase (DXS, MEP pathway) in chloroplasts; geranyl PP synthase (GPPS), geranylgeranyl PP synthase (GGPPS), and geranylgeranyl PP reductase (GGPPR) in both chloroplasts and the oil body membrane; farnesyl diphosphate synthase (FPPS) in the cytoplasm (the oil body membrane is absent from the chloroplast); and monoterpene synthase (MTPS) in both the chloroplast and the lumen of oil bodies. This evidence suggests that terpenoid biosynthesis takes place in the cytosol, the chloroplasts, and the oil bodies, with some of the enzymes being localized in more than one organelle [28].

A typical plant terpene synthase (TPS) catalyzes terpenoid formation in seed plants. Terpenoid biosynthesis in non-seed plants has gradually been explored in recent genomic and transcriptomic studies, revealing deeper insights into terpenoid biosynthesis in these plants. Typical plant synthase genes are absent in non-seed plants. The evolution of TPS genes in early land plants opens the door to investigating the diversity of terpenoids in seed plants [75]. MPTSLs, i.e., microbial terpene synthase-like genes, are responsible for the terpenoid diversity in non-seed plants. These enzymes are phylogenetically and structurally more similar to bacterial and fungal terpene synthases than to typical plant terpene synthases. They are present in non-seed plants but absent in plants and green algae [76]. Microbial TPs from bacteria and fungi are only distantly related to typical plant TPSs, whereas genes similar to microbial TPSs have been recently identified in *Selaginella moellendorffii*. MPTSL proteins from non-seed plants form four major groups, two of which are more closely related to bacterial TPSs and the other two to fungal TPSs. Two of the four groups contain a canonical aspartate-rich “DDxxD” motif. The third group has a “DDxxxD” motif, and the fourth group has conserved only the first two “DD” in this motif. Each of the four groups displayed catalytic functions as monoterpene and sesquiterpene synthases, suggesting that these are important for terpene formation in non-seed plants [75].

Seventeen putative terpene synthase genes from *M. polymorpha* transcriptomes were identified. Of these, four diterpene synthase genes are phylogenetically related to those found in diverged plants, and nine unusual monoterpene and sesquiterpene synthase-like genes are present. Mono- and sesquiterpene synthases represent a distinct class of enzymes, not related to previously described plant terpene synthases and only distantly related to microbial-type terpene synthases. The absence of a Mg^2+^-binding, aspartate-rich, DDXXD motif places these enzymes in a non-canonical family of terpene synthases [73]. Seven full-length MTPSL genes were identified in *Anthoceros punctatus* (AP MTPSL 1–7) based on the analysis of its genome sequence. Thirteen *Anthoceros* MTPSL genes were cloned into a protein expression vector. E. coli expressing recombinant MTPSLs from hornworts were assayed for terpene synthase activities. All functional MTPSLs exhibited sesquiterpene synthase activities [57]. The bryophyte *Physcomitrella patens* has a single TPS gene, copalyl synthase/kaurene synthase (CPS/KS), encoding a bifunctional enzyme producing ent-kaurene, which is a precursor of gibberellins. The genome of the lycophyte *Selaginella moellendorffii* contains 18 TPS genes, and the genomes of some model angiosperms and gymnosperms contain 40–152 TPS genes, not all of which are functional, with most of the functional ones having lost activity in either the CPS- or KS-type domains. TPS genes are generally divided into seven clades, with some plant lineages having a majority of their TPS genes in one or two clades, indicating the lineage-specific expansion of specific types of genes [77].

TPSs are important enzymes responsible for terpenoid diversity. Investigating the differentiation of the TPS gene family could provide valuable theoretical support for the genetic involvement of oil-producing plants. While the origin and function of TPS genes have been extensively studied, the exact origin of the initial gene fusion event that occurred in plants or microbes remains uncertain. Phylogenetic analysis revealed that the fusion of the TPS gene likely occurred in the ancestor of land plants following the acquisition of individual C- and N-terminal domains. Potential mutual transfer of the TPS gene was observed among microbes and plants. Gene synteny analysis disclosed a differential divergence pattern between TPS-c and TPS-e/f subfamilies involved in primary metabolism and TPS-a/b/d/g/h subfamilies crucial for secondary metabolites [78].

## 6. Conclusions

This up-to-date literature study reveals the exploration of the chemical diversity of bryophyte taxa from around the globe. A study of the last few decades shows the emergence of genetic engineering and genomic studies, as discussed in Section 5 regarding the terpenoid biosynthesis study and the characterization of specific enzymes and their evolution. Exploration of the genomic studies of the last few decades shows that bryophytes have emerged as promising tools for plant metabolic engineering due to their rapid transformation and haploid-dominant life cycle. *Marchantia polymorpha* and *Physcomitrella patens* are the best-studied, and genetic studies for both species allow for efficient pathway engineering. Bryophytes have a rich profile of terpenoid accumulation, which may be facilitated by flux modes in central metabolism that support high rates of withdrawal of the appropriate carbon skeletons, energy, and reducing power. It follows that the best production host for this class of compounds may be one in which such flux modes already exist. This includes species that already accumulate high amounts of terpenoids [79].

Five genes involved in artemisinin biosynthesis were engineered into the moss *Physcomitrella* patens via direct in vivo assembly of multiple DNA fragments. In vivo biosynthesis of artemisinin was obtained without further modifications. *P. patens* is a sustainable and efficient production platform of artemisinin that, without further modifications, can be used for industrial-scale production [80]. *Physcomitrella patens* is an ideal platform for diterpene engineering because of its efficient genome-editing capability and the absence of endogenous diterpenoids after targeted disruption of its native diterpene synthase. Heterologous co-expression of modular diterpene synthases in *Physcomitrella patens* leads to the accumulation of diterpenoids found in contemporary land plants [81]. *Marchantia paleacea* L. has emerged as a promising chassis for synthetic biology applications. Patchoulol production has been investigated by engineering its biosynthetic pathway, and compartmentalized metabolic engineering has proven to be an effective strategy for enhancing sesquiterpene production [82].

Recent advances in genomic, transcriptomic, and metabolomic research will provide future researchers with valuable resources for applying genome editing to the targeted production of specific metabolites. Model bryophytes such as *Marchantia polymorpha* and *Physcomitrella patens* have the potential to serve as next-generation biotechnological chassis for the industrial-scale production of valuable terpenoids.

## Figures and Tables

**Table 1:** Example of some terpenoid compounds detected from liverworts and mosses used for chemosystematics.

Species/Genus/Family/ Order Name	Terpenoid Class	Major Compounds	References
*Frullania* sp.	sesquiterpenoid and aromatics	sesquiterpenoid-lactone and bibenzyl	[37]
Jungermanniales *Aneura* (Metzgeriales)	sesquiterpenoid	pinguisane type	[4]
*Pellia endiviifolia* (Metzgeriales)	diterpenoid	sacculatane	[4]
*Pallavicinia levieri* (Metzgeriales)	diterpenoid	sacculatane	[4]
*Riccardia robusta* var. *yakushimensis* (Metzgeriales)	diterpenoid	sacculatane	[4]
*Fossombronia alaskana* (Metzgeriales)	diterpenoid	sacculatane	[4]
*Bazzania* sp.	sesquiterpenoid	bazzanane and cuparane	[46]
*Riccardia lobata* var. *yakushimensis*	diterpenoid	sacculatane-type	[47]
*Riccardia crassa*	diterpenoid	sacculatane-type	[47]
*Pleurozia gigantea*	diterpenoids	labdane-, clerodane-, and fusicoccane	[5]
*Chandonanthus hirtellus*	diterpenoid	cembranes	[5]
*Pallavicinia* species	diterpenoids	7,8-secolabdane	[5]
*Conocephalum conicum* species A	phenylpropanoid ester (O)	(E) methylcinnamate	[48]
*Conocephalum conicum* species F	sesquiterpenoid	cyclocolorenone	[48]
*Conocephalum conicum* species J	monoterpene hydrocarbon	sabinene	[48]
*Conocephalum conicum* species L	sesquiterpene alcohol	conocephalenol	[48]
*Conocephalum salebrosum (=Conocephalum conicum* species S)	sesquiterpenoid	cubebol	[48]
*Porella* sp. (I)	sesquiterpenoid	Drimane	[49]
*Porella* sp. (II)	diterpenoid	sacculatane	[49]
*Porella* sp. (III)	sesquiterpenoid–diterpenoid	pinguisane–sacculatane	[49]
*Porella* sp. (IV)	sesquiterpenoid	guaiane–germacrane	[49]
*Porella* sp. (V)	sesquiterpenoid	pinguisane	[49]
*Porella* sp. (VI)	sesquiterpenoid	africane	[49]
*Hypnum plumaeforme* (M)	diterpenoid	primarane (momilactone A)	[42].
*Hypnum plumaeforme* (M)	diterpenoid	primarane (momilactone B)	[42].

Notes: M = moss; the rest of the taxa are liverworts. Apart from terpenoids, other classes of compounds are denoted by (O).

**Table 2:** Some of the compounds responsible for characteristic odor of liverworts.

Species Name	Compound Class	Compound	Odor Property	References
*Plagiochila lutilans*	monoterpenoids	terpinene, terpinolene, limonene, p-cymene, *β*-phellandrene, p-cymen-8-ol, pulegone, 3,7-dimethyl-2,6-octadien-1,6-olide, menthone, isomenthone, sabinene, and b-pinene, among which pulegone was the major component. Additional abundant components were terpinolene, limonene, and p-cymen-8-ol.	peppermint-like	[50]
*Plagiochila standleyi*	monoterpenoid	p-cymene, b-phellandrene, and ascaridole, limonene	peppermint	[50]
*Plagiochila killarniensis*, *P. spinulosa*, and *P. punctata*	monboterpene hydrocarbon	*β*-phellandrene	strong aromatic smell when fresh species are crushed	[50]

## Data Availability

No new data were created or analyzed in this study. Data sharing is not applicable to this article.
